# A Weakly Supervised Deep Learning Model and Human–Machine Fusion for Accurate Grading of Renal Cell Carcinoma from Histopathology Slides

**DOI:** 10.3390/cancers15123198

**Published:** 2023-06-15

**Authors:** Qingyuan Zheng, Rui Yang, Huazhen Xu, Junjie Fan, Panpan Jiao, Xinmiao Ni, Jingping Yuan, Lei Wang, Zhiyuan Chen, Xiuheng Liu

**Affiliations:** 1 Department of Urology, Renmin Hospital of Wuhan University, Wuhan 430060, China; zqy710890394@whu.edu.cn (Q.Z.); drwanglei@whu.edu.cn (L.W.); 2 Institute of Urologic Disease, Renmin Hospital of Wuhan University, Wuhan 430060, China; 3 Department of Pharmacology, School of Basic Medical Sciences, Wuhan University, Wuhan 430072, China; 4 University of Chinese Academy of Sciences, Beijing 100049, China; 5 Trusted Computing and Information Assurance Laboratory, Institute of Software, Chinese Academy of Sciences, Beijing 100190, China; 6 Department of Pathology, Renmin Hospital of Wuhan University, Wuhan 430060, China

**Keywords:** clear cell renal cell carcinoma, tumor grading, deep learning, whole slide image, human–machine fusion

## Abstract

**Simple Summary:**

Renal cell carcinoma causes over 179,000 deaths per year worldwide, and the Fuhrman grading (FG) system is crucial for diagnosing this deadly cancer. However, visual histopathological assessment is influenced by inter-observer variability and irreproducibility. In this study, we trained a deep learning model named SSL-CLAM using whole slide histopathology images to objectively diagnose the FG status of patients with clear cell renal cell carcinoma (ccRCC). We demonstrated that the SSL-CLAM model successfully diagnosed five FG states of ccRCC (Grade-0, 1, 2, 3, and 4) and validated the results in two independent cohorts. The attention heatmap of the SSL-CLAM model visualized high attention regions, and we found that cell nuclear size, contour, and cellular pleomorphism were critical morphologies that align with the existing FG criteria. In summary, a human–machine collaborative diagnostic model may assist pathologists in making diagnostic decisions, and further prospective clinical trials are needed to confirm its efficacy.

**Abstract:**

(1) Background: The Fuhrman grading (FG) system is widely used in the management of clear cell renal cell carcinoma (ccRCC). However, it is affected by observer variability and irreproducibility in clinical practice. We aimed to use a deep learning multi-class model called SSL-CLAM to assist in diagnosing the FG status of ccRCC patients using digitized whole slide images (WSIs). (2) Methods: We recruited 504 eligible ccRCC patients from The Cancer Genome Atlas (TCGA) cohort and obtained 708 hematoxylin and eosin-stained WSIs for the development and internal validation of the SSL-CLAM model. Additionally, we obtained 445 WSIs from 188 ccRCC eligible patients in the Clinical Proteomic Tumor Analysis Consortium (CPTAC) cohort as an independent external validation set. A human–machine fusion approach was used to validate the added value of the SSL-CLAM model for pathologists. (3) Results: The SSL-CLAM model successfully diagnosed the five FG statuses (Grade-0, 1, 2, 3, and 4) of ccRCC, and achieved AUCs of 0.917 and 0.887 on the internal and external validation sets, respectively, outperforming a junior pathologist. For the normal/tumor classification (Grade-0, Grade-1/2/3/4) task, the SSL-CLAM model yielded AUCs close to 1 on both the internal and external validation sets. The SSL-CLAM model achieved a better performance for the two-tiered FG (Grade-0, Grade-1/2, and Grade-3/4) task, with AUCs of 0.936 and 0.915 on the internal and external validation sets, respectively. The human–machine diagnostic performance was superior to that of the SSL-CLAM model, showing promising prospects. In addition, the high-attention regions of the SSL-CLAM model showed that with an increasing FG status, the cell nuclei in the tumor region become larger, with irregular contours and increased cellular pleomorphism. (4) Conclusions: Our findings support the feasibility of using deep learning and human–machine fusion methods for FG classification on WSIs from ccRCC patients, which may assist pathologists in making diagnostic decisions.

## 1. Introduction

Renal cell carcinoma (RCC) is the most common renal tumor, affecting approximately 4.4 individuals per 100,000 people globally [1,2]. According to The International Agency for Research on Cancer, there were 431,288 new cases and 179,368 deaths in 2020 [3]. The most common histological subtype of RCC is clear cell RCC (ccRCC), which accounts for 90% of cases and has a poor prognosis [4]. The Fuhrman grading (FG) system and tumor–node–metastasis (TNM) staging system are important clinical evidence for assessing the malignancy and predicting the prognosis of ccRCC [5,6]. FG is based on the evaluation of nuclear features, including nuclear size, shape, and prominence of nucleoli [7]. Based on these evaluations, ccRCC is classified into one of four different grades (Grade-1, 2, 3, or 4). Although the International Society of Urological Pathology has introduced a new ccRCC grading system, which has been incorporated into the renal tumor classification system by the World Health Organization, FG is still widely used in clinical management [8]. Generally, pathologists diagnose and grade RCC by examining histological images of the tumor. However, this manual process is time consuming, and there is a risk of misdiagnosis or missed diagnoses, with previously reported inter-observer variability [9,10]. Therefore, there is an urgent need for a rapid, objective, and accurate cancer grading diagnostic system to address the challenge.

Artificial intelligence technology has become one of the most promising fields in computational pathology [11,12,13,14]. Multiple studies have shown that deep learning algorithms can extract key features from hematoxylin and eosin (H&E)-stained histopathological images, enabling diagnosis and subtyping with comparable or better accuracy than expert pathologists [15,16,17,18,19]. This evidence not only demonstrates the potential of deep learning to improve traditional diagnosis and prediction, but also helps pathologists reduce repetitive and tedious work, freeing up time to handle more complex tasks [20]. Hence, we assume that deep learning can facilitate the grading diagnosis of ccRCC and improve traditional diagnostic methods. However, most deep learning models rely heavily on manual pixel-level annotations, which seriously hinders the development of artificial intelligence in computational pathology. It is necessary to further use unsupervised or weakly supervised models to improve clinical applicability while ensuring model performance.

Image-driven deep learning has the potential to improve the accuracy of visual diagnosis. In ccRCC patients, H&E-stained slides have some morphological features that can be identified by pathologists, including tumor cell clusters and extensive lymphocyte infiltration [21,22]. Pathologists can learn and utilize these known features to stage or grade patients. In addition to these recognizable features, deep learning may extract more morphological features that pathologists are not yet aware of, leading to more accurate diagnoses.

In this study, we used a weakly supervised multi-class deep learning model [23] with a self-supervised learning (SSL) feature extractor, named SSL-CLAM, to accurately diagnose the FG status of ccRCC from H&E-stained whole slide images (WSIs). We validated the robustness of diagnostic model in two independent cohorts and revealed the interpretability of the model. Importantly, we further demonstrated a human–machine fusion strategy that utilizes the SSL-CLAM model to assist pathologists in making diagnoses, which can simultaneously improve the diagnostic performance of both parties.

## 2. Materials and Methods

### 2.1. Patient Cohorts

**Figure 1:**
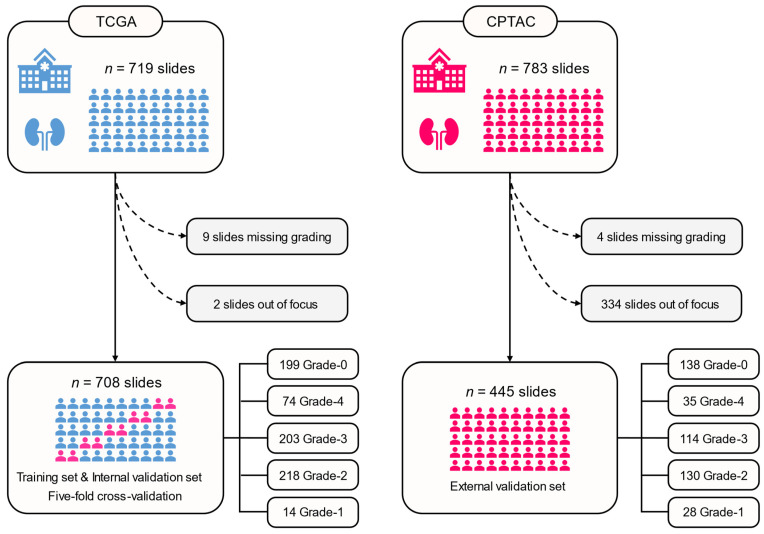
The recruitment pathway of this study. Available patients and whole slide images were recruited from the TCGA and CPTAC cohorts. TCGA, The Cancer Genome Atlas; CPTAC, Clinical Proteomic Tumor Analysis Consortium.

In this study, we developed SSL-CLAM by retrospectively analyzing two independent cohorts. The first cohort was The Cancer Genome Atlas (TCGA) dataset, which included 519 H&E-stained WSIs from 513 candidate ccRCC patients. After excluding cases with poor pathological image quality, or missing FG information or important clinical information, a total of 509 WSIs were included in the analysis. Additionally, we collected 199 H&E-stained images of normal tissues for Grade-0. The second cohort was from the Clinical Proteomic Tumor Analysis Consortium (CPTAC) dataset, consisting of 783 H&E-stained WSIs from 222 candidate ccRCC patients. After excluding cases with poor pathological image quality, or missing FG information or important clinical information, a total of 445 WSIs were included in the analysis, of which 307 were tumors and 138 were normal tissues. Furthermore, we collected all available clinical and pathological information of the patients in both cohorts and confirmed informed consent. The WSIs and clinico-pathological information of the TCGA cohort can be downloaded directly from the NIH GDC data portal of the TCGA-KIRC project, while those of the CPTAC cohort can be downloaded from the CPTAC data portal. The recruitment pathway is illustrated in Figure 1.

### 2.2. WSI Preprocessing

First, foreground and background segmentation were performed on all WSIs to identify the tissue regions (foreground). The binary mask of tissue regions was computed by thresholding the saturation channel of the median blurred image to smooth edges, followed by morphological closing to fill small gaps and holes. After segmentation, patches were extracted from the tissue regions of each WSI at 20× magnification, with a size of 256 × 256 pixels. Each patch and its corresponding information were stored in the hdf5 data format. A pretrained ResNet-50 model based on SSL was used to extract 1024-dimensional feature vectors from each patch, which were cropped after the third residual block.

### 2.3. Deep Learning Algorithm

This study utilized the clustering-constrained-attention multiple-instance learning (CLAM) framework [23] for implementation. CLAM is a weakly supervised deep learning method that employs an attention-based multiple-instance learning model to automatically detect subregions of high diagnostic value, enabling accurate classification of the entire WSI. It also incorporates instance-level clustering to restrict and refine the feature space. CLAM employs an attention-based pooling function to aggregate patch-level features into slide-level representations for classification. Additionally, the model identifies and ranks all patches within the tissue region, assigning an attention score to each patch to indicate its contribution or importance in the slide-level prediction.

Unlike conventional binary classification algorithms that focus on positive/negative categorization, CLAM is designed to address multi-class problems. The CLAM model incorporates N parallel attention branches, which collectively generate N distinct slide-level predictions. Each prediction is derived from a distinct set of high-attention regions within the WSI. The classification layer assesses the class-specific slide predictions to derive the overall slide prediction probability.

**Figure 2:**
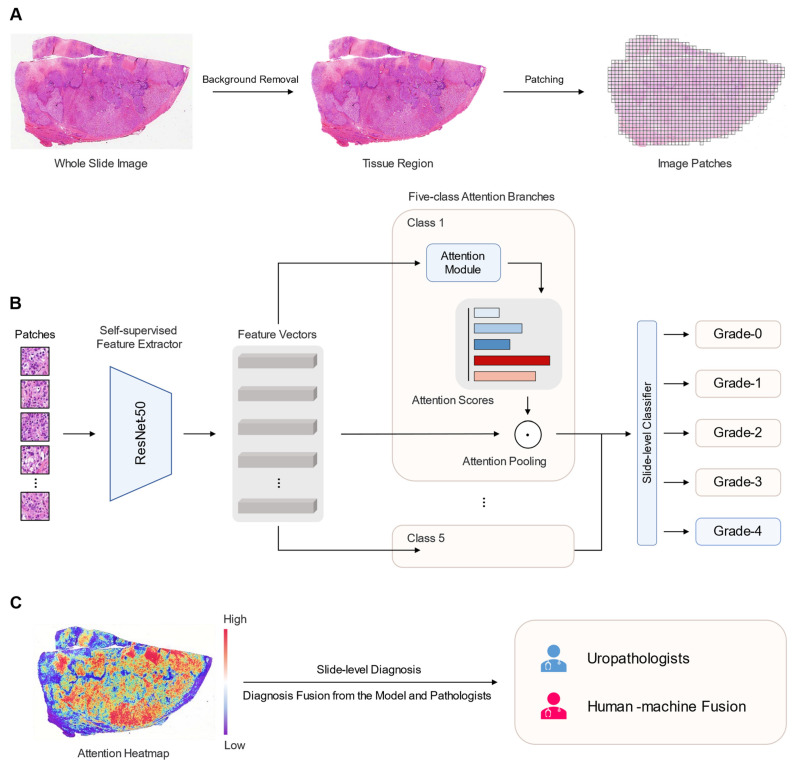
Schematic workflow of this study. (**A**) Preprocessing of whole slide images. All whole slide images have their backgrounds removed and divided into small patches (256 × 256 pixels) to fit the convolutional neural network architecture. (**B**) Pretrained ResNet-50 based on self-supervised learning for feature extraction. For multi-classification tasks, attention-based multi-instance learning is applied. (**C**) Attention heatmap is used to explain deep learning model and improve diagnostic performance with human–machine fusion mode.

Most previous works, including those using CLAM, have primarily concentrated on using pretrained weights from the ImageNet dataset. However, recent research has demonstrated that image feature extractors (encoders) can be pretrained via self-supervised learning (SSL) [24]. This approach has shown improved performance compared to relying on the ImageNet weights. Wang et al. [25] employed a clustering-guided contrastive SSL algorithm to train a ResNet-50 model using a vast collection of 15 million pathology images extracted from 32,000 WSIs sourced from the TCGA and PAIP datasets. Therefore, here, we used the pretrained ResNet-50 with these weights as the feature extractor for CLAM, named SSL-CLAM. In this study, SSL-CLAM was used to solve a five-classification task, targeting the five grades of ccRCC: normal/non-cancerous (Grade-0), Grade-1, Grade-2, Grade-3, and Grade-4. Specifically, when a WSI is input for prediction, SSL-CLAM will output predicted probability values corresponding to the five different categories. The probability values range from 0 to 1, and their sum is equal to 1. The TCGA dataset was randomly divided into a training set and an internal validation set in a 4:1 ratio, and the model was trained using a five-fold cross-validation strategy. The CPTAC dataset was used for external evaluation of SSL-CLAM. The schematic workflow of this study is shown in Figure 2.

### 2.4. Human–Machine Fusion

In order to further evaluate the performance of the SSL-CLAM model, we invited a junior pathologist A undergoing training, and a chief expert uropathologist B, to jointly diagnose 445 WSIs from the CPTAC cohort. They were blinded to the labels and clinico-pathological information of these WSIs beforehand. First, they independently made diagnoses for each WSI relying on their own expertise, and repeated the review five times. Subsequently, with the aid of heatmaps and slide-level diagnoses of SSL-CLAM, five replicate diagnostics were performed. The results of each diagnosis were recorded and compared with the performance of SSL-CLAM.

### 2.5. Interpretability of the Model

To better understand the morphological features used by the deep learning model for classification, we visualized interpretable heatmaps with normalized attention scores using the attention module of SSL-CLAM. In these heatmaps, regions with high attention scores are shown in red and contribute more to decisions made by the model, while regions with low attention scores are shown in blue and contribute less to decisions made by the model.

### 2.6. Statistical Analysis

The classification performance of the SSL-CLAM model was evaluated using receiver operating characteristic curves and area under the curve (AUC), as well as accuracy and precision. For all AUC, accuracy, and precision calculations, we used the macro-average evaluation method. Cohen’s kappa coefficient was calculated to evaluate the diagnostic consistency between the SSL-CLAM model and pathologists/human–machine fusion methods. McNemar’s test was performed to compare the diagnostic differences in accuracy between the SSL-CLAM model and pathologists/human–machine fusion methods. Differences were considered significant when the *p* value from the two-tailed test was <0.05. Python (version 3.8.13) and the deep learning platform PyTorch (version 1.10) were used for model construction and data analysis.

## 3. Results

### 3.1. Patient Characteristics

We used 708 WSIs from 504 ccRCC patients in the TCGA cohort for the development and internal validation of the SSL-CLAM model, including 199 Grade-0, 14 Grade-1, 218 Grade-2, 203 Grade-3, and 74 Grade-4 WSIs. Considering the limited sample size of Grade-0, which may lead to a decrease in model performance, we adopted a data augmentation strategy for Grade-1 and obtained 70 Grade-1 WSIs. Additionally, we used 445 WSIs from 188 ccRCC patients in the CPTAC cohort for external validation of the SSL-CLAM model, including 138 Grade-0, 28 Grade-1, 130 Grade-2, 114 Grade-3, and 35 Grade-4 WSIs. Table 1 summarizes the demographic and clinico-pathological characteristics of the two cohorts, and the detailed data distribution is shown in Supplementary Table S1.

### 3.2. Diagnostic Performance of the SSL-CLAM Model

**Figure 3:**
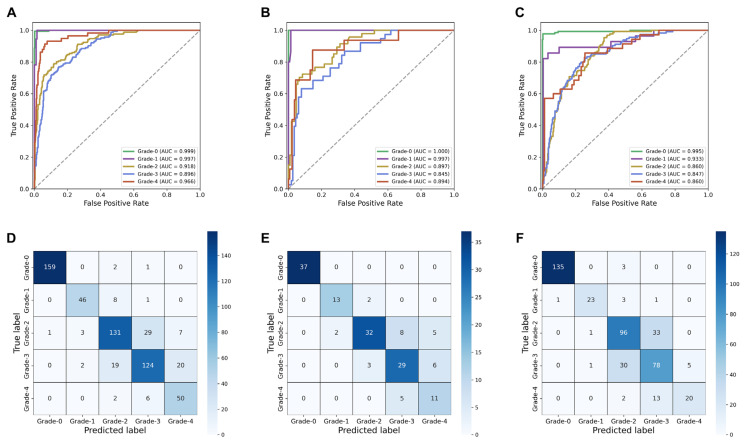
Receiver operating characteristic curves of the SSL-CLAM model for five-class Fuhrman grading task in the (**A**) training set, (**B**) internal validation set, and (**C**) external validation set. (**D**), (**E**), and (**F**) show the confusion matrices of the training set, internal validation set, and external validation set, respectively.

Firstly, in the five-class classification task (Grade-0, 1, 2, 3, 4), the SSL-CLAM model yielded an average accuracy and AUC of 0.818 (95% confidence interval [CI], 0.805–0.831) and 0.947 (95% CI, 0.938–0.956) on the training set, respectively. On the internal validation set, the SSL-CLAM model yielded an average accuracy and AUC of 0.776 (95% CI, 0.742–0.812) and 0.917 (95% CI, 0.905–0.928), respectively. Even on the external validation set, the SSL-CLAM model demonstrated strong generalization ability, with an average accuracy and AUC of 0.771 (95% CI, 0.739–0.803) and 0.887 (95% CI, 0.872–0.904), respectively. Figure 3 shows the receiver operating characteristic curves and confusion matrices of the highest-performing SSL-CLAM model. In the internal validation set, all 37 Grade-0 cases were correctly predicted as Grade-0. Thirteen of the fifteen Grade-1 cases were correctly predicted as Grade-1, but two were predicted as Grade-2. Two of the forty-seven Grade-2 cases were predicted as Grade-1, eight were predicted as Grade-3 and the other five were predicted as Grade-4. Three of the thirty-eight Grade-3 cases were predicted as Grade-2, and six were predicted as Grade-4. Of the 16 Grade-4 cases, only 11 were correctly predicted. The misdiagnoses included 5 cases predicted as Grade-3. Overall, 122 (79.9%) of the 153 cases were correctly predicted in the internal validation set, and 352 (77.4%) of the 455 cases were correctly predicted in the external validation set.

**Figure 4:**
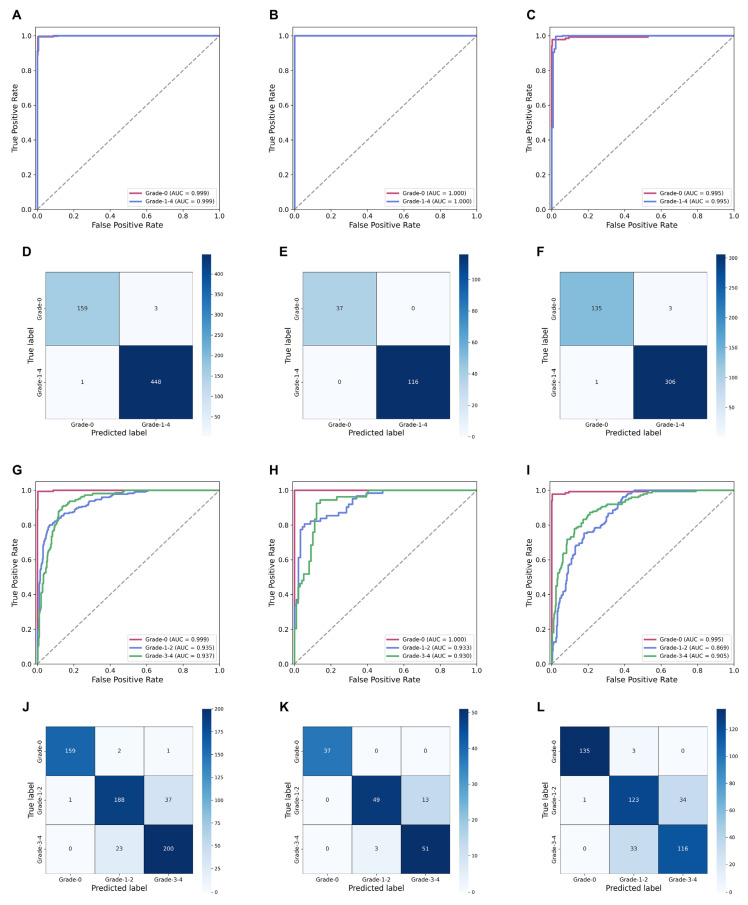
Receiver operating characteristic curves of the SSL-CLAM model for normal/tumor classification task in the (**A**) training set, (**B**) internal validation set, and (**C**) external validation set. (**D**,**E**) and (**F**) show the confusion matrices of normal/tumor classification task in the training set, internal validation set, and external validation set, respectively. Receiver operating characteristic curves of the SSL-CLAM model for two-tiered Fuhrman grading task in the (**G**) training set, (**H**) internal validation set, and (**I**) external validation set. (**J**,**K**,**L**) show the confusion matrices of two-tiered Fuhrman grading task in the training set, internal validation set, and external validation set, respectively.

In the task of binary classification (Grade-0, Grade-1/2/3/4), we obtained a robust cancer diagnostic model using the SSL-CLAM model. The average accuracy and AUC on the internal validation set were 0.997 (95% CI, 0.992–1.000) and 0.999 (95% CI, 0.999–1.000), respectively, and on the external validation set they were 0.989 (95% CI, 0.986–0.992) and 0.991 (95% CI, 0.989–0.994), respectively (Table 2 and Figure 4A–F).

Furthermore, we investigated the diagnostic performance of the SSL-CLAM model on a simplified two-tiered FG system. The FG merged Grade-1 and Grade-2 as low-grade tumors and Grade-3 and Grade-4 as high-grade tumors, simplifying the four-tiered grading system into two tiers. The average accuracy and AUC using the internal validation set were 0.872 (95% CI, 0.845–0.899) and 0.936 (95% CI, 0.906–0.962), respectively, and on the external validation set they were 0.838 (95% CI, 0.829–0.847) and 0.915 (95% CI, 0.907–0.922), respectively (Table 2 and Figure 4G–L).

In addition, we invited a junior pathologist A, who is undergoing training, and a chief expert uropathologist B to jointly diagnose 445 WSIs from the CPTAC cohort. As shown in Table 3, the accuracy of junior pathologist A was 0.737 (95% CI; 0.721–0.753), while our grading diagnostic model SSL-CLAM outperformed A (*p* = 0.002, paired chi-square test). The diagnostic accuracy of expert uropathologist B was 0.824 (95% CI; 0.808–0.839). There was no significant difference between SSL-CLAM and expert B (*p* > 0.05, paired chi-square test). The SSL-CLAM model achieved good interobserver agreement with expert uropathologist B (kappa = 0.889) (Table 3).

### 3.3. Human–Machine Fusion

To investigate whether the SSL-CLAM model could help pathologists in diagnosis in clinical practice, we further employed a human–machine fusion strategy to test the performance of two pathologists (A and B) with the assistance of the SSL-CLAM model. The diagnostic fusion of SSL-CLAM with junior pathologist A and expert uropathologist B achieved an average accuracy of 0.787 (95% CI; 0.772–0.801) and 0.856 (95% CI; 0.843–0.867), respectively, which were superior to the use of SSL-CLAM alone (Table 3). Both pathologists showed improved diagnostic performance with the assistance of the SSL-CLAM model.

### 3.4. Attention-Based Interpretation Analysis

**Figure 5:**
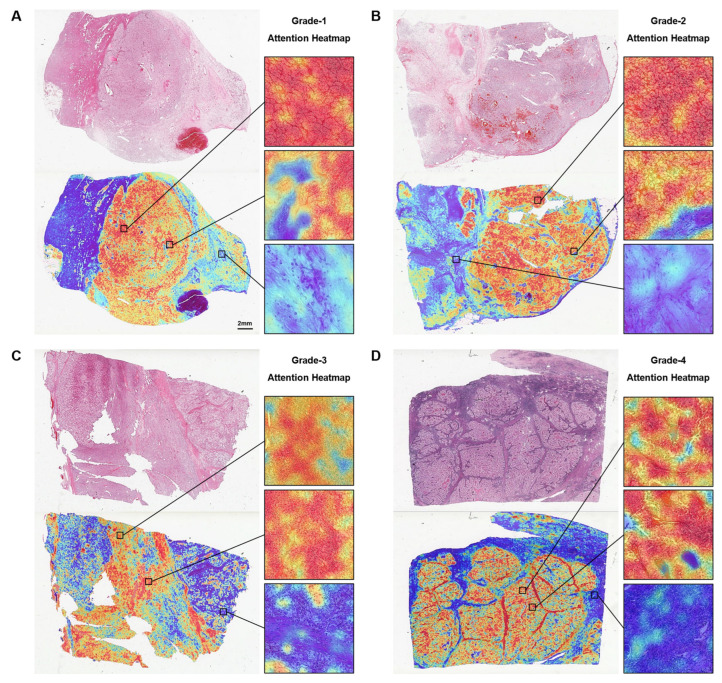
Representative whole slide images and attention heatmaps of Fuhrman grading for (**A**) Grade-1, (**B**) Grade-2, (**C**) Grade-3, and (**D**) Grade-4.

Next, we used the highest-performing model during the training process to conduct attention-based visualization analysis, in order to reveal the black-box property of the deep learning model and understand the morphological features that contribute the most to the FG diagnosis. Figure 5 displays the attention heatmaps of the model for the four FG statuses. Overall, the heatmaps showed a higher focus on dense and deeply stained tumor cells, and lower focus on surrounding normal tissues (Figure 5). As the FG grade increased, the size of the tumor cell nuclei that the model paid attention to became significantly enlarged at the same magnification. In the Grade-1 tumor area, the tumor cell nuclei were relatively uniform and regular, similar to normal cell nuclei. In the Grade-2 and Grade-3 tumor areas, the nuclei began to exhibit irregular contours. This phenomenon became more pronounced in Grade-4, with more dysplastic cells appearing.

## 4. Discussion

In this study, we used two independent cohorts to confirm that the deep learning model SSL-CLAM can accurately diagnose the FG status in ccRCC H&E-stained WSIs with high performance. Specifically, SSL-CLAM outperformed a junior pathologist and achieved good inter-observer agreement with an expert uropathologist. Importantly, our study further demonstrated that a human–machine fusion strategy can improve the performance of SSL-CLAM in diagnosing FG status, although further prospective clinical trials are needed to confirm its effectiveness.

A recent study showed that a deep learning-based, fully automated classifier can diagnose FG statuses with an accuracy of 90.09% [26]. However, their method requires pathologists to manually annotate all billion pixels of the slides, which would severely hinder its clinical applicability. More importantly, there was no comparison of diagnostic performance between the deep learning model and pathologists. Whether a deep learning model can provide additional value to current clinical pathologists in diagnosing FG status is still unknown. Our study is the first to compare the performance of a deep learning model with that of pathologists in the diagnosis of FG in ccRCC. The performance of the SSL-CLAM model was significantly better than that of a junior pathologist and is expected to exceed that of an expert pathologist. In the human–machine fusion, the diagnostic performance of the human–machine fusion was better than that of SSL-CLAM, suggesting that inexperienced junior pathologists can combine the diagnosis of SSL-CLAM with their own diagnosis to achieve an overall expert-level diagnostic performance.

The most widely used predictive method in computational pathology is to patch images from digitized WSIs to train a multi-instance learning model [27,28,29]. Lu et al. [23] proposed the CLAM model based on a gated attention method, which aggregates patch-level features and subsequently aggregates the information required for multi-classification at the slide level, making it more suitable for diagnosing FG status. A significant advantage of CLAM is that it uses an attention mechanism to aggregate patch-level instances into slide-level predictions, generating interpretable heatmaps. In addition, training CLAM only requires slide-level labels without the need for additional annotations, thereby improving data utilization efficiency and reducing a significant amount of manual annotation. Most importantly, we used ResNet-50 pretrained on SSL as the image feature extractor, which has been demonstrated to outperform traditional pretrained methods based on ImageNet [24,25].

The most widely used nuclear grading scheme in the world is the FG system; however, the inter-observer consistency for diagnosis using the traditional four-tiered FG is moderate [30]. This is attributed to RCC being a heterogeneous tumor, typically composed of cells of different grades rather than all cells being of the same grade [31]. It has been shown that the simplified two-tiered FG (Grade-1 combined Grade-2, Grade-3 combined Grade-4) performs as well as the traditional four-tiered FG. Using the simplified grading scheme may be more advantageous for pathologists and clinicians [32,33]. From a clinical practice perspective, pathologists do not need to distinguish between Grade-1 and Grade-2 tumors as their difference lies in the presence of nucleoli only visible at 400× magnification. Similarly, the difference between Grade-3 and Grade-4 tumors lies in the presence of bizarre multinucleated cells [32]. This relatively subjective definition may undermine the reproducibility of FG. In this study, we also analyzed the two-tiered FG using SSL-CLAM. The results showed that SSL-CLAM had a better diagnostic performance for the two-tiered FG compared to the four-tiered FG. It appears that breaking down the FG system into a lower grade (Grade-1 and Grade-2) group and higher grade (Grade-3 and Grade-4) group may better match clinical use.

Deep learning models are often considered “black boxes”, as their working mechanisms are not transparent. To explain the diagnostic pattern of SSL-CLAM, we used attention heatmaps to determine the diagnostic features used by SSL-CLAM. We observed significant differences in the nuclei of tumor cells within the highly focused regions of the model. Specifically, the nuclei of low-FG tumor cells exhibited uniformity, and unclear and irregular contours, while the nuclei of high-FG tumor cells showed distinct irregular contours and a higher presence of atypical cells. This diagnostic pattern is consistent with the grading criteria proposed by Fuhrman et al. [34]: Grade-1 tumors are characterized by small nuclei (~10 μm) that are round, uniform, with indistinct or absent nucleoli; Grade-2 tumors have larger (~15 μm) nuclei with irregular contours and nucleoli visible at high magnification (400×); Grade-3 tumors have even larger nuclei (~20 μm) with distinct irregular contours and prominent nucleoli, visible even at low magnification (100×); Grade-4 tumors exhibit similar features to Grade-3 tumors but also have bizarre, often multinucleated cells and heavy chromatin clumping. Thus, an advantage of this study is that heatmaps were created to aid in the visualization of the regions of interest, making it easier to understand the basic principles underlying the decision-making process of SSL-CLAM.

Although SSL-CLAM has yielded promising results, our study does have some limitations. First, further prospective clinical trials are needed to confirm the effectiveness of SSL-CLAM and the human–machine fusion strategy. Second, the unbalanced number of FGs in the TCGA cohort will affect the performance of SSL-CLAM. More available data needs to be collected in the future to eliminate the negative impact of class imbalance. Third, we observed that the fusion of the expert uropathologist with the SSL-CLAM model significantly enhanced the accuracy of SSL-CLAM. Comparing the assistance provided to pathologists by clinico-pathological information rather than relying solely on SSL-CLAM is worth further exploration. Four, we observed the presence of artifacts and some interfering factors in some slides that met the inclusion criteria in both cohorts. Although the attention of SSL-CLAM is focused less on these areas according to the visualization, it is necessary to establish a standardized procedure for pathological slide image creation to improve the quality of images in the future.

## 5. Conclusions

We investigated the FG status of ccRCC diagnosed from H&E-stained WSIs using SSL-CLAM. The attention heatmaps provide a visualization of high-attention areas at the cellular level, allowing for the analysis of potential factors used in the pathological diagnoses. In addition, the diagnostic performance of SSL-CLAM is apparently not as reliable as that of an expert uropathologist but may be superior to that of inexperienced pathologists; however, this remains to be determined. A human–machine fusion diagnostic mode may help pathologists make diagnostic decisions, and further prospective clinical trials are needed to confirm its effectiveness.

## Figures and Tables

**Table 1:** Clinico-pathological features of TCGA and CPTAC cohorts.

	TCGA	CPTAC
Number of patients	504	188
WSI format	SVS	SVS
Age (years)	60.57 (±12.20)	60.92 (±12.05)
Gender		
Female	177 (35.12%)	64 (34.04%)
Male	327 (64.88%)	124 (65.96%)
pT stage		
pT1	257 (50.99%)	58 (30.85%)
pT2	66 (13.10%)	14 (7.45%)
pT3	170 (33.73%)	41 (21.8%)
pT4	11 (2.18%)	3 (1.6%)
pTx	0 (0%)	72 (38.3%)
pN stage		
pN0	230 (45.63%)	23 (12.23%)
pN1	14 (2.78%)	4 (2.13%)
pNx	260 (51.59%)	161 (85.64%)
pM stage		
pM0	402 (79.76%)	33 (17.55%)
pM1	74 (14.68%)	3 (1.6%)
pMx	28 (5.56%)	152 (80.85%)
pTNM stage		
Stage I	251 (49.81%)	84 (44.68%)
Stage II	54 (10.71%)	20 (10.64%)
Stage III	118 (23.41%)	47 (25.00%)
Stage IV	80 (15.87%)	21 (11.17%)
Missing	1 (0.20%)	16 (8.51%)
Fuhrman grade		
G1	12 (2.38%)	13 (6.92%)
G2	216 (42.86%)	95 (50.53%)
G3	202 (40.08%)	60 (31.91%)
G4	74 (14.68%)	20 (10.64%)
Survival status		
Alive	334 (66.27%)	145 (77.13%)
Dead	170 (33.73%)	27 (14.36%)
Not reported	0 (0%)	16 (8.51%)
Overall survival (years)	3.64 (± 2.67)	2.43 (± 1.83)

**Table 2:** Accuracy and AUC of the SSL-CLAM model.

**a. Diagnostic Performance in Five-Class Fuhrman Grade (Grade-0, 1, 2, 3, 4)**		
	Accuracy (95% CI)	AUC (95% CI)
Training set	0.818 (0.805, 0.831)	0.947 (0.938, 0.956)
Internal validation set	0.776 (0.742, 0.812)	0.917 (0.905, 0.928)
External validation set	0.771 (0.739, 0.803)	0.887 (0.872, 0.904)
**b. Diagnostic performance in normal/tumor classification (Grade-0, Grade-1/2/3/4)**		
	Accuracy (95% CI)	AUC (95% CI)
Internal validation set	0.997 (0.992, 1.000)	0.999 (0.999, 1.000)
External validation set	0.989 (0.986, 0.992)	0.991 (0.989, 0.994)
**c. Diagnostic performance in two-tiered Fuhrman grading (Grade-0, Grade-1/2, Grade-3/4)**		
	Accuracy (95% CI)	AUC (95% CI)
Internal validation set	0.872 (0.845, 0.899)	0.936 (0.906, 0.962)
External validation set	0.838 (0.829, 0.847)	0.915 (0.907, 0.922)

**Table 3:** Comparisons of the SSL-CLAM model with human pathologists and human–machine fusion in the external validation set.

	Accuracy (95% CI)	Precision (95% CI)	*p*-Value *	Kappa #
SSL-CLAM model	0.771 (0.739, 0.803)	0.786 (0.762, 0.811)	-	-
Junior Pathologist A	0.737 (0.721, 0.753)	0.695 (0.678, 0.712)	0.002	0.837
Expert Uropathologist B	0.824 (0.808, 0.839)	0.800 (0.779, 0.821)	0.336	0.889
Junior A—SSL-CLAM fusion	0.787 (0.772, 0.801)	0.773 (0.758, 0.788)	0.902	0.904
Expert B—SSL-CLAM fusion	0.856 (0.843, 0.867)	0.839 (0.819, 0.858)	<0.001	0.906

* A paired chi-square test (McNemar’s test) was used to examine differences in accuracy between the SSL-CLAM model and each uropathologist/human–machine fusion; # Inter-observer agreement between the SSL-CLAM model and each uropathologist/human–machine fusion assessed by the Cohen kappa coefficient.

## Data Availability

The datasets of the TCGA cohort for this study can be found in The Cancer Genome Atlas Program (https://portal.gdc.cancer.gov/, accessed on 1 March 2023) and the CPTAC cohort for this study can be found in the Clinical Proteomic Tumor Analysis Consortium (https://www.cancerimagingarchive.net/histopathology-imaging-on-tcia/, accessed on 1 March 2023).

## Supplementary Materials

The following supporting information can be downloaded at: https://www.mdpi.com/article/10.3390/cancers15123198/s1, Table S1a: Dataset distribution of patients and corresponding images in the SSL-CLAM model; Table S1b: Dataset distribution of images in the training, internal validation, and external validation sets.
