# Supplementary material for: A Weakly Supervised Deep Learning Model and Human–Machine Fusion for Accurate Grading of Renal Cell Carcinoma from Histopathology Slides
Source: Cancers (Basel). 2023 Jun 15;15(12):3198. doi: 10.3390/cancers15123198 (PMC10296233; doi:10.3390/cancers15123198)
Supplement: Supplementary file 1 [file cancers-15-03198-s001.zip › cancers-2412917-supplementary.pdf]

Supplementary Materials

Supplementary Table S1a. Dataset distribution of patients and corresponding images in the SSL-CLAM model.

	Patients	Total images	Grade-0 images	Grade-1 images	Grade-2 images	Grade-3 images	Grade-4 images
TCGA Cohort	504	764	199	14/70*	218	203	74
CPTAC Cohort	188	445	138	28	130	114	35

* The number of Gade-1 images from TCGA after data argumentation.

Supplementary Table S1b. Dataset distribution of images in the training, internal validation, and external validation sets.

	TCGA Cohort		CPTAC Cohort
	Training set	Internal validation set	External validation set
SSL-CLAM	611	153	445
